# Nonsurgical treatment for asymptomatic pancreatolithiasis is meaning: A case report

**DOI:** 10.1097/MD.0000000000031557

**Published:** 2022-10-28

**Authors:** Satoshi Yamamoto, Kazuo Inui, Yoshiaki Katano, Hironao Miyoshi, Takashi Kobayashi, Yoshihiko Tachi, Masashi Hattori

**Affiliations:** a Department of Gastroenterology, Fujita Health University Bantane Hospital, Nagoya, Japan; b Department of Gastroenterology, Yamashita Hospital, Ichinomiya, Japan; c Department of Gastroenterology, Fujita Health University Okazaki Medical Center, Okazaki, Japan.

**Keywords:** abdominal pain, asymptomatic pancreatolithiasis, chronic pancreatitis

## Abstract

Many guidelines for nonsurgical treatment of pancreatolithiasis suggest little guidance for patients with pancreatolithiasis who do not have abdominal pain. Some patients with pancreatolithiasis whom we have treated nonsurgically with extracorporeal shock-wave lithotripsy did not have abdominal pain, and we describe one of them here.

**Methods and Results:**

A 42-year-old man complaining of an 8-kg weight loss over 6 months was admitted to a nearby hospital, where fasting blood sugar and hemoglobin A1c values were 500 mg/dL and 11.8%. Computed tomography showed stones in the head of the pancreas and dilation of the main pancreatic duct. He was referred to our hospital to be considered for nonsurgical treatment of pancreatolithiasis. His height and weight were 160 cm and 52 kg (body mass index, 20.31). No tenderness or other abdominal findings were evident. After obtaining informed consent for nonsurgical treatment despite absence of abdominal pain, we performed extracorporeal shock wave lithotripsy. Computed tomography showed disappearance of stones from the pancreatic head. At discharge, his weight had increased to 62 kg and hemoglobin A1c was 6.8%, though antidiabetic medication has since become necessary.

**Conclusion:**

We believe that nonsurgical treatment of pancreatolithiasis was helpful for this patient, and could improve exocrine and endocrine function in other patients without abdominal pain.

## 1. Introduction

Many guidelines for nonsurgical treatment of pancreatolithiasis with abdominal pain have been published by various medical specialty societies,^[1–6]^ but these suggestions offer little guidance for nonsurgical treatment of patients with pancreatolithiasis who do not have abdominal pain. Some of the 165 patients with pancreatolithiasis whom we have treated nonsurgically with extracorporeal shock-wave lithotripsy between 1992 and 2020^[7]^ did not have abdominal pain, and we describe one of them here. The patient has provided informed consent for publication of the case, and the patient’s identity has been protected.

## 2. Case presentation

**Figure 1.**
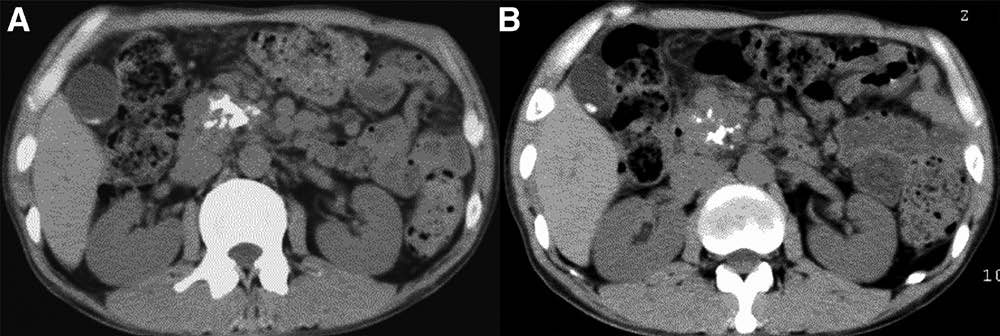
CT findings before and after nonsurgical treatment for pancreatolithiasis without abdominal pain. (A) Before nonsurgical treatment, CT showed stones with molded contours and diameters up to 20 mm in the duct within the pancreatic head. B. After nonsurgical treatment, CT showed disappearance of stones from the pancreatic head. CT = computed tomography.

A 42-year-old man complaining of an 8-kg weight loss over 6 months was admitted to a nearby hospital, where fasting blood sugar and hemoglobin (Hb) A1c values were 500 mg/dL and 11.8%. Computed tomography (CT) showed stones in the head of the pancreas and dilation of the main pancreatic duct. He was referred to our hospital to be considered for nonsurgical treatment for pancreatolithiasis. He had consumed 60 g of ethanol daily for 20 years and 20 cigarettes a day for 10 years and was hospitalized twice for acute pancreatitis. He denied present or recent abdominal pain. On physical examination, his height was 160 cm, his weight was 52 kg (body mass index, 20.31), and he showed no tenderness or other abdominal abnormalities. On admission, the serum pancreatic amylase concentration was low (9 IU/L), and an N-benzoyl-L-tyrosyl-p-aminobenzoic acid test result (39.9%) confirmed exocrine pancreatic dysfunction. CT performed on admission showed multiple stones in the main pancreatic duct within the pancreatic head. These showed molded contours and diameters up to 20 mm (Fig. 1). Proximally to the stones the duct was dilated, and the pancreatic body and tail were atrophic. Radiography with abdominal compression confirmed presence of radiopaque stones in the pancreatic head; subsequent endoscopic retrograde pancreatic cannulation confirmed multiple proximally located defects within the contrast material filling the main duct.

We attributed weight loss and worsening of diabetes to stone impaction, expecting significant recovery of pancreatic function upon treatment because functional decline had been rapid and brief. We therefore obtained informed consent for extracorporeal shock-wave lithotripsy despite absence of abdominal pain. After 9 extracorporeal shock-wave lithotripsy procedures, CT showed disappearance of stones from the pancreatic head and decreased proximal dilation of the duct (Fig. 1). After additional endoscopic treatment, endoscopic retrograde pancreatic cannulation showed complete clearance of stones.

**Figure 2.**
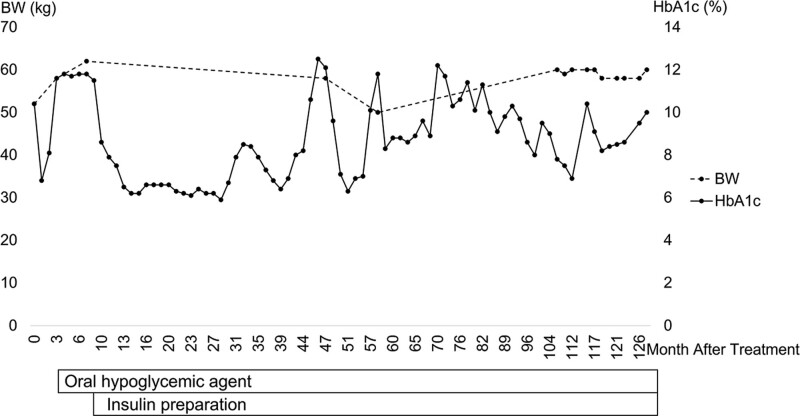
Clinical course.

After nonsurgical treatment, weight increased to 62 kg. Control of diabetes was maintained by diet alone at the time of discharge, and CT had improved to 6.8%. However, HbA1c increased again 2 months after treatment, and the patient required an oral hypoglycemic agent. By 8 months after treatment, he additionally required insulin injections, and his diabetes now is managed by a specialist at a nearby hospital. His weight has remained near 60 kg, and HbA1c fluctuations appear related to transient impaction of protein plugs in the main pancreatic duct (Fig. 2). Stones have not recurred.

## 3. Discussion and conclusion

The international consensus guidelines for chronic pancreatitis published in 2020 do not recommend endoscopic treatment for asymptomatic patients.^[6]^ Before these guidelines were published we had performed nonsurgical treatment for 165 patients with pancreatolithiasis between 1992 and 2020.^[7]^ Abdominal pain was absent in 41, while 124 had abdominal pain. Both of these groups showed preservation of pancreatic exocrine function, with N-benzoyl-L-tyrosyl-p-aminobenzoic acid test results of about 50%; these results were maintained during the year following treatment. Nonsurgical treatment of pancreatolithiasis therefore appears to preserve pancreatic exocrine function whether or not patients present with abdominal pain.^[8]^ In the present patient, we are uncertain about the durability of exocrine function preservation by treatment since we did not repeat the N-benzoyl-L-tyrosyl-p-aminobenzoic acid test during follow-up. Considering endocrine function, however, his post-treatment HbA1c value and body weight showed improvement even though not all of that progress was maintained, and more aggressive diabetes management was required. If the patient had not wanted nonsurgical treatment, we suspect that increasingly serious worsening of diabetes and body weight would have continued. We therefore believe that overall, nonsurgical treatment was helpful for this patient, and that it will prove to be worthwhile in preserving pancreatic function in many similar patients.

## Acknowledgments

The authors would like to thank all study members and the patient.

## Author contributions

All authors have read and approved the manuscript.

**Conceptualization:** Kazuo Inui, Yoshiaki Katano.

**Data curation:** Masashi Hattori.

**Investigation:** Satoshi Yamamoto.

**Project administration:** Kazuo Inui, Yoshiaki Katano, Hironao Miyoshi, Takashi Kobayashi, Yoshihiko Tachi, Masashi Hattori.

**Resources:** Satoshi Yamamoto.

**Writing—original draft:** Satoshi Yamamoto.

## References

[1] Hoffmeister A Mayerle J Beglinger C; members of the guideline committee. English language version of the S3-consensus guidelines on chronic pancreatitis: definition, aetiology, diagnostic examinations, medical, endoscopic and surgical management of chronic pancreatitis. Z Gastroenterol. 2015;53:1447–95. 26666283 10.1055/s-0041-107379

[2] Dumonceau JM Delhaye M Tringali A. Endoscopic treatment of chronic pancreatitis: European Society of Gastrointestinal Endoscopy (ESGE) guideline- updated August 2018. Endoscopy. 2019;51:179–93. 30654394 10.1055/a-0822-0832

[3] Frulloni L Falconi M Gabbrielli A; Italian Association for the Study of the Pancreas (AISP). Italian consensus guidelines for chronic pancreatitis. Dig Liver Dis. 2010;42:S381–406. 21078490 10.1016/S1590-8658(10)60682-2

[4] Ito T Ishiguro H Ohara H. Evidence-based clinical practice guidelines for chronic pancreatitis 2015. J Gastroenterol. 2016;51:85–92. 26725837 10.1007/s00535-015-1149-x

[5] Dominguez-Munoz JE Drewes AM Lindkvist B; HaPanEU/UEG Working Group. HaPanEU/UEG Working Group. Recommendations from the United European Gastroenterology evidence-based guidelines for the diagnosis and therapy of chronic pancreatitis. Pancreatology. 2018;18:847–54. 30344091 10.1016/j.pan.2018.09.016

[6] Kitano M Gress TM Garg PK. International consensus guidelines on interventional endoscopy in chronic pancreatitis. Recommendations from the working group for the international consensus guidelines for chronic pancreatitis in collaboration with the International Association of Pancreatology, the American Pancreatic Association, the Japan Pancreas Society, and European Pancreatic Club. Pancreatology. 2020;20:1045–55. 32792253 10.1016/j.pan.2020.05.022

[7] Yamamoto S Inui K Katano Y. Pancreatic stones: clinical outcomes with nonsurgical treatment in a Japanese single-center study. Pancreas. 2022;51:205–11. 35404899 10.1097/MPA.0000000000001996

[8] Yamamoto S Inui K Katano Y. Benefit from nonsurgical treatment for asymptomatic pancreatolithiasis. Pancreas. 2022;51:510–5. 35835109 10.1097/MPA.0000000000002062

